# Spontaneous labor curve based on a retrospective multi‐center study in Japan

**DOI:** 10.1111/jog.15053

**Published:** 2021-10-07

**Authors:** Ryosuke Shindo, Shigeru Aoki, Toshihiro Misumi, Sayuri Nakanishi, Takeshi Umazume, Takeshi Nagamatsu, Hisashi Masuyama, Atsuo Itakura, Tomoaki Ikeda, Japan Society of Obstetrics and Gynecology Perinatal Committee

**Affiliations:** ^1^ Perinatal Center for Maternity and Neonates Yokohama City University Medical Center Yokohama Japan; ^2^ Department of Biostatistics Yokohama City University Graduate School of Medicine Yokohama Japan; ^3^ Department of Obstetrics and Gynecology Hokkaido University Graduate School of Medicine Sapporo Japan; ^4^ Department of Obstetrics and Gynecology, Faculty of Medicine The University of Tokyo Tokyo Japan; ^5^ Department of Obstetrics and Gynecology Okayama University Graduate School of Medicine, Dentistry and Pharmaceutical Sciences Okayama Japan; ^6^ Department of Obstetrics and Gynecology Juntendo University Faculty of Medicine Tokyo Japan; ^7^ Department of Obstetrics and Gynecology Mie University School of Medicine Mie Japan

**Keywords:** active phase, first stage of labor, Friedman curve, labor curve, spontaneous labor

## Abstract

**Aim:**

In Japan, the criteria of the latent and active phases of the first stage of labor have not been decided. The Japan Society of Obstetrics and Gynecology (JSOG) Perinatal Committee conducted a study to construct a spontaneous labor curve in order to determine the point of onset of the active phase.

**Methods:**

The participants were women who had spontaneous deliveries at four health facilities in Japan between September 1, 2011, and September 31, 2019. Spontaneous delivery was defined as the spontaneous onset of labor at term (37 weeks, 0 days to 41 weeks, 6 days) with vaginal delivery of a mature fetus in a cephalic position without uterotonic agents or epidural analgesia. The time points for each “cm” of dilation were collected starting from the time of full dilation retrogradely. The relationship between time since labor onset and cervical dilation was expressed as a curve using a smoothing B‐spline.

**Results:**

A total of 4215 primiparous and 5266 multiparous women were included in this study. The spontaneous labor curve showed that in both primiparous and multiparous women, labor progress was slow until 5 cm cervical dilation, accelerating between 5 and 6 cm dilation, and steadily progressed after 6 cm dilation.

**Conclusion:**

We propose that the active phase of the first stage of labor be defined as starting at 5 cm dilation of the cervix, and that it be divided into an acceleration phase (5–6 cm dilation) and a maximal phase (>6 cm dilation).

## Introduction

The first stage of labor is divided into a latent phase, characterized by a slow cervical dilation rate, and an active phase, with an accelerated progression of labor. If the progression of labor is slower than the standard, interventions such as oxytocin augmentation are considered. Proposed in the 1950s, the Friedman curve has been widely used in Japan as the standard progression.^1^, ^2^ According to the Friedman curve, the latent phase occurs up to 3–4 cm of cervical dilation, after which, the active phase follows.^3^ However, it is a labor curve created from only 500 primiparous women, and it is questionable whether it can be applied to modern pregnant women. In 2010, Zhang et al.^4^ developed a new, contemporary labor curve in the United States using data from 62 415 women. Based on this labor curve, the American College of Obstetricians and Gynecologists (ACOG) redefined the onset of the active phase to be at 6 cm of cervical dilation.^5^ Subsequently, reports on labor curves sprouted from numerous countries, allowing the World Health Organization (WHO)^6^ to define the onset of the active stage as 5 cm of cervical dilation based on three reviews.^7^, ^8^, ^9^ In Japan, Suzuki et al. developed a labor curve based on data from approximately 2000 primiparous women and reported that it was similar to Zhang's curve.^10^ However, all these previously reported labor curves do not reflect the progression of spontaneous labor, as they include cases that utilized uterotonic agents and analgesia.

Both the ACOG^5^, ^11^ and the Japanese Society of Obstetrics and Gynecology (JSOG) guidelines^12^ agree that the latent phase of labor is highly individualized; therefore, interventions such as oxytocin augmentation are not always necessary. In Japan, the onset of the active phase is not clearly defined, and there is a possibility that excessive and unnecessary labor augmentations are being performed. Inappropriate use of uterotonic agents can lead to the development of maternal complications such as uterine rupture and birth canal laceration, as well as serious fetal adverse events such as cerebral palsy. In light of this, we, the JSOG Perinatal Committee, aimed to evaluate the natural progression of labor and to define the onset of the active phase by constructing a labor curve from normal, spontaneous term labors with no medical interventions, such as oxytocin augmentation or epidural analgesia.

## Methods

In this retrospective, multi‐center study, data were collected from existing medical records. The study was conducted as an initiative of the JSOG with the approval of the Ethics Committee of Yokohama City University (approval no. B191200016). The subjects of this study were primiparous and multiparous women who had spontaneous deliveries at four health facilities (three university hospitals and one tertiary referral hospital) in Japan between September 1, 2011, and September 31, 2019. Spontaneous delivery was defined as the spontaneous onset of labor at term (37 weeks, 0 days to 41 weeks, 6 days) and the vaginal delivery of a mature fetus in a cephalic position without the use of interventions such as uterotonic agents or epidural analgesia, with exception of routine episiotomies. Since the progression of delivery differs greatly between primiparous and multiparous women, these were analyzed separately. Maternal characteristics such as age at delivery, height, pre‐pregnancy and delivery weight, BMI, gestational age at delivery, infant birthweight, and infant sex were collected. Data related to the progress of labor were collected, as follows: (1) the time of hospital admission for the onset of labor; (2) the degree of cervical dilation upon admission; (3) the degree of cervical dilation at a certain time, as evaluated using internal examination by a physician or midwife; (4) the time of delivery; and (5) the number of internal examinations before the cervix reached full dilation.

The period between admission for the onset of labor and full dilation of the cervix was defined as the time required for the first stage of labor. The period between full dilation of the cervix to the delivery of the baby was defined as the time required for the second stage of labor. The time of full cervical dilation was set as the baseline, and the time points before full cervical dilation were collected retrogradely for each cm of dilation. The degree of cervical dilation (from 0 to 10 cm) was plotted on the vertical axis against time on the horizontal axis to create a scatter plot. In addition, the relationship between time and cervical dilation was expressed as a curve using a smoothing B‐spline for primiparous and multiparous women. The degree of the B‐spline function was determined using the Akaike information criterion (AIC) method. The time required for the cervix to open by 1 cm was calculated using an inverse estimation approach based on the estimated labor curve for each degree of opening on the labor curve. We used a bootstrap method with 3000 replications to calculate 95% confidence intervals (CI).

## Results

There was a total of 9481 eligible patients, of whom, 4215 were primiparous women and 5266 were multiparous women. Maternal characteristics are shown in Table 1. The median age at delivery was 31 years for primiparous women and 34 years for multiparous women. The median gestational age was 39 weeks for both groups, and the median birthweight of the newborns was 2988 g for primiparous women and 3064 g for multiparous women.

**TABLE 1:** Participant characteristics

	Primiparous women, median (IQR) *n* = 4215	Multiparous women, median (IQR) *n* = 5267
Age, years old	31 (28–35)	34 (30–37)
Height, cm	158 (156–163)	159 (155–162)
Pre‐pregnancy weight, kg	51 (47–56)	52 (48–57)
Weight at delivery, kg	62 (57–67.8)	63 (58–69)
Pre‐pregnancy BMI, kg/m^2^	20.2 (18.8–21.7)	20.4 (19.0–22.5)
BMI at delivery, kg/m^2^	24.2 (22.7–26.2)	24.7 (23.0–26.9)
Gestational weeks at delivery, weeks	39 (38–40)	39 (38–40)
Infant birthweight, g	2988 (2768–3230)	3064 (2826–3312)

Abbreviation: IQR, interquartile range.

Table 2 shows the time required for delivery and the number of internal examinations performed for primiparous and multiparous women until delivery. The median time for the first stage of labor for primiparous women was 7.8 h, and the 95th percentile value was 21.0 h. The median time to delivery was 8.4 h, and the 95th percentile value was 23.2 h. On the other hand, the median time required for the first stage of labor for multiparous women was 4.0 h, and the 95th percentile value was 12.0 h. The median time to delivery was 4.4 h, and the 95th percentile was 12.7 h. Thus, multiparous women delivered at half the time of primiparous mothers.

**TABLE 2:** Time required for each stage of delivery and number of internal examinations

	Primiparous women *n* = 4215	Multiparous women *n* = 5267
Duration of the first stage (hours): median (5–95 percentile)	7.8 (1.8–21.0)	4.0 (1.0–12.0)
Duration of the second stage (hours): median (5–95 percentile)	1.0 (0.2–4.2)	0.2 (0.0–1.4)
Duration of labor (hours): median (5–95 percentile)	8.4 (2.8–23.2)	4.4 (1.3–12.7)
Number of internal examinations: median (IQR)	4 (3–6)	3 (2–5)

Abbreviation: IQR, interquartile range.

**FIGURE 1:**
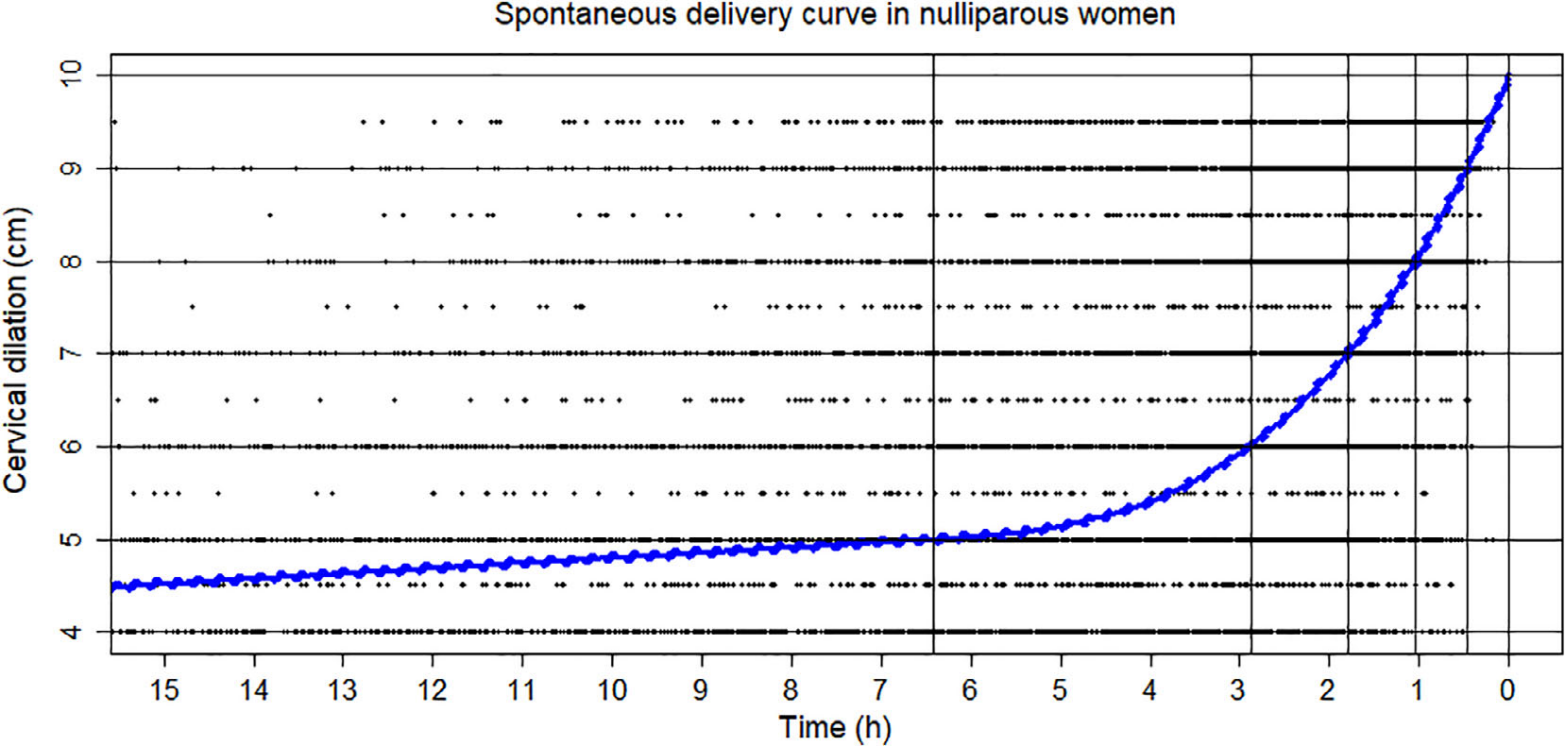
The spontaneous labor curve of primiparous women. It was constructed from 4215 primiparous women who had a spontaneous delivery. The degree of cervical dilation (from 0 to 10 cm) was plotted on the vertical axis against time on the horizontal axis to create a scatter plot. The relationship between time and cervical dilation was expressed as a curve using a smoothing B‐spline

**FIGURE 2:**
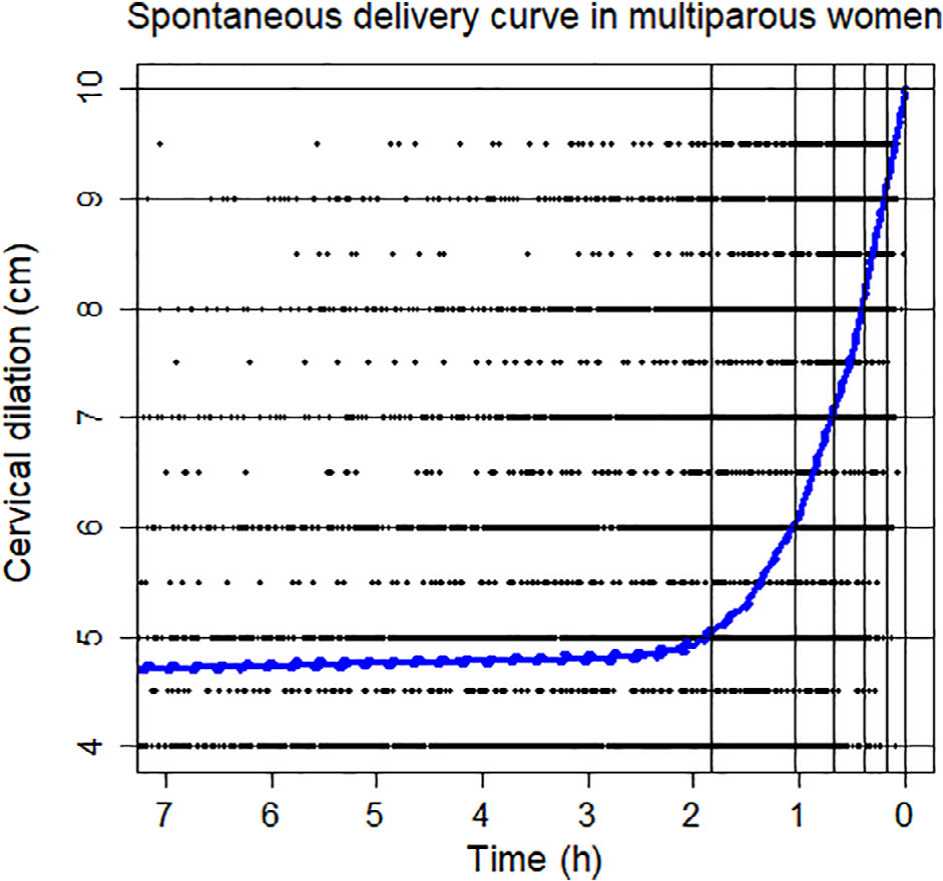
The spontaneous labor curve of multiparous women. It was constructed from 5266 multiparous women who had a spontaneous delivery. The degree of cervical dilation (from 0 to 10 cm) was plotted on the vertical axis against time on the horizontal axis to create a scatter plot. The relationship between time and cervical dilation was expressed as a curve using a smoothing B‐spline

**FIGURE 3:**
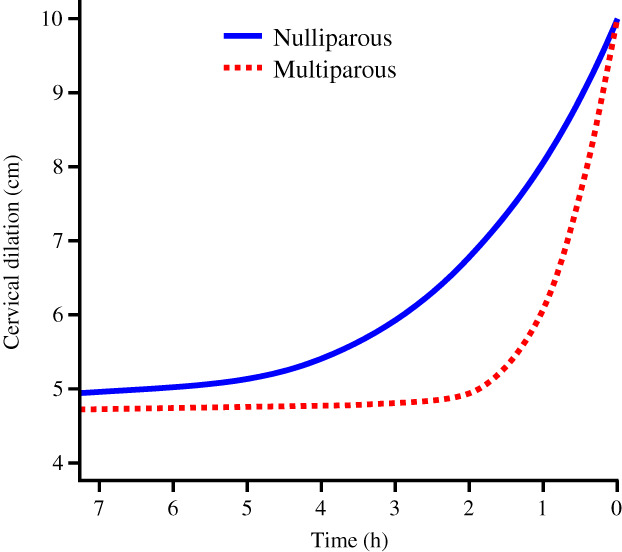
Comparison of the spontaneous labor curves of primiparous and multiparous women

Figure 1 shows the labor curve of primiparous women. The slope was initially gentle, and the progression of labor was slow until the cervical dilation was 5 cm. After dilation of the cervix at 5 cm, progression accelerated. The mean time needed to progress was 3.55 h for 5–6 cm, and 2.88 h for 6–10 cm (Table 3). Thus, delivery accelerated at 5 cm of cervical dilation and progressed more rapidly from 6 cm. Figure 2 shows the labor curve of multiparous women. The curve was almost flat until the cervical dilation was 5 cm, but the slope changed after 5 cm. The mean time needed to progress was 0.79 h for 5–6 cm, and 1.05 h for 6–10 cm (Table 3). The delivery progressed more rapidly after 6 cm. Figure 3 shows a comparison of the labor curves of primiparous and multiparous women. It is obvious that the labor progresses more rapidly in multiparous women.

**TABLE 3:** Time required to dilate the cervix by 1 cm

Cervical dilation	Primiparous women (h), mean (95% confidence interval)	Multiparous women (h), mean (95% confidence interval)
5–6 cm	3.55 (5.17)	0.79 (0.73–0.98)
6–7 cm	1.09 (1.34)	0.38 (0.37–0.41)
7–8 cm	0.75 (0.85)	0.28 (0.27–0.29)
8–9 cm	0.58 (0.60)	0.22 (0.21–0.22)
9–10 cm	0.46 (0.49)	0.17 (0.16–0.19)
6–10 cm	2.88 (N/A)	1.05 (N/A)

## Discussion

**FIGURE 4:**
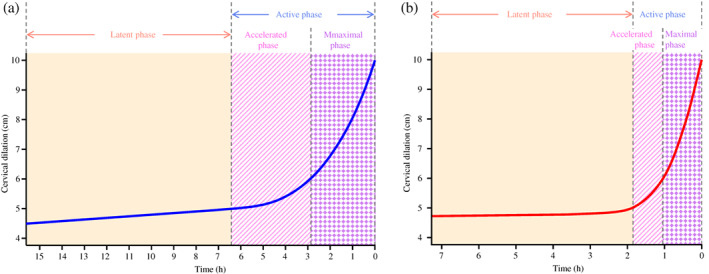
Phases of the first stage of labor in (a) primiparous and (b) multiparous women from the spontaneous labor curve. The active phase of the first stage of labor is dilation of the cervix after 5 cm and that the active phase is further divided into the accelerated phase (dilation of the cervix 5–6 cm) and the maximal phase (dilation of the cervix after 6 cm)

In this study, the first stage of labor for deliveries without medical interventions, such as uterotonic agents or epidural anesthesia, was illustrated as a spontaneous labor curve. The spontaneous labor curve was different in shape from either Friedman's^1^, ^2^, ^3^, ^13^ or Zhang's^4^ curve (Figures S1 and S2). The spontaneous labor curve showed that in both primiparous and multiparous women, the progression of the first stage of labor was almost horizontal until cervical dilation at 5 cm, accelerating between 5 and 6 cm, and demonstrating a steady upturn after 6 cm. Based on the results of this study, we propose that the active phase of the first stage of labor is defined as dilation of the cervix after 5 cm and that the active phase is further divided into the accelerated phase (dilation of the cervix 5–6 cm) and the maximal phase (dilation of the cervix after 6 cm) (Figure 4).

In the Friedman curve, the active phase of the first stage of labor is subdivided into three phases: acceleration, maximal slope, and deceleration phases. The acceleration phase is a short period that bridges the latent phase and the maximal. The maximal slope phase is the period in which labor progresses most quickly, with cervical dilation ranging from about 3–4 cm to 8–9 cm. The deceleration phase is the period from 9 cm dilation to full dilation. On the other hand, the curve of Zhang et al.^4^ did not make these subdivisions but simply grouped together the period after 6 cm of cervical dilation as the active phase.

At 3–4 cm of cervical dilation, which was already in the active phase in the Friedman curve, the spontaneous labor curve in this study was almost horizontal and the progress of delivery was very slow. Furthermore, in the Friedman curve, there is a deceleration phase in which the progression slows down when the cervical dilation is 9–10 cm, but no deceleration phase was observed in the spontaneous labor curve. As pointed out by Zhang et al.,^4^ these differences may be because the number of patients analyzed in the construction of the Friedman curve was small, the curve was created almost 50 years ago, and the method of constructing the curve was different.

In comparison with Zhang et al.'s^4^ curve, the spontaneous labor curve was quite similar, but certain differences were also found. In the spontaneous labor curve, the active phase was considered to be divided into the accelerated phase and the maximal phase. For primiparous women, the time required for dilating the cervix from 5 to 6 cm was 3.55 h for the spontaneous labor curve and 0.8 h for Zhang et al.'s curve, notably longer for the subjects in this study. Furthermore, when the progression of labor after 6 cm was compared, the time required to dilate the cervix by 1 cm was almost constant in the results of Zhang et al., whereas in this study, this period became shorter as the delivery progressed. For multiparous women, the time required to dilate the cervix from 5 to 6 cm was 0.79 h for the spontaneous labor curve, and 0.8 h for the curve developed by Zhang et al., it was almost the same. However, when the progression of labor after 6 cm was compared, the time required to dilate the cervix by 1 cm was shorter than that reported by Zhang et al. Similar to primiparous women, the period decreased as the delivery progressed (Tables 3 and 4). The reasons for these differences can be explained as follows. First, more than 40% of the subjects in Zhang et al.’s study received oxytocin augmentation and approximately 80% received epidural anesthesia, whereas the subjects in our study did not receive these medical interventions. Second, in contrast to Zhang et al., who constructed a labor curve starting from the time of admission to the hospital, the spontaneous labor curve was constructed from the time of full dilation of the cervix for only those labors that progressed smoothly (without the need for intervention), which resulted in a smaller variation in the time in active labor.

**TABLE 4:** Time required to dilate the cervix in Zhang's curve

	Primiparous (h), median (95th percentile)	Parity = 1 (h), median (95th percentile)	Parity = 2 or more (h), median (95th percentile)
4–5 cm	1.3 (6.4)	1.4 (7.3)	1.4 (7.0)
5–6 cm	0.8 (3.2)	0.8 (3.4)	0.8 (3.4)
6–7 cm	0.6 (2.2)	0.5 (1.9)	0.5 (1.8)
7–8 cm	0.5 (1.6)	0.4 (1.3)	0.4 (1.2)
8–9 cm	0.5 (1.4)	0.3 (1.0)	0.3 (0.9)
9–10 cm	0.5 (1.8)	0.3 (0.9)	0.3 (0.8)

There is only one report that analyzed a population without oxytocin augmentation or analgesia similar to the present study.^13^ ^)^ The investigators constructed a labor curve for a total of 3172 primiparous and multiparous mothers who delivered at one university hospital and six primary medical facilities. The curve suggested that the onset of the active stage of labor was at 6 cm cervical dilation in primiparous women and 5 cm cervical dilation in multiparous women. On the other hand, unlike our study, their report was limited to cases with maternal age of 20–39 years, BMI of less than 30, and infant birthweight of appropriate for dates. However, it is notable that their insights on the shape of the curve and the starting point of the active phase were generally consistent with the results of the present study.

A strength of this study is the reduced variation in delivery time due to the retrospective examination of only deliveries with a smooth course. It is also favorable that the active phase could be further divided into the accelerated phase and the maximal phase, unlike the curve of Zhang et al.^4^ ^)^ In addition, by collecting data from multiple university hospitals, we were able to reduce policy bias among institutions. However, this study also has several limitations. First, the participating facilities are biased toward tertiary referral hospital, and second, the duration and frequency of internal examinations are not based on a uniform protocol because of the retrospective nature of the study. Related to this, the criteria for oxytocin augmentation were also not standardized. Therefore, it cannot be denied that only patients with short labor times were included in the study, especially if oxytocin augmentation was performed at an earlier time. Further studies considering with the perinatal outcomes at various facilities including the maternity houses are needed.

In conclusion, in spontaneous labor without medical intervention, the active phase began at 5 cm of cervical dilation and labor progressed more rapidly after 6 cm in both primiparous and multiparous women. We, the JSOG Perinatal Committee, propose that the active phase of the first stage of labor is defined as cervical dilation after 5 cm and that the active phase is further divided into an accelerated phase (5–6 cm of cervical dilation), in which the progression of labor begins to accelerate, and a maximal phase (after 6 cm of cervical dilation), in which labor progresses more rapidly.

## Disclosure

The authors have no conflicts of interest to disclose.

## Author Contributions

Ryosuke Shindo wrote the initial draft of the manuscript. Sayuriu Nakanishi assisted writing the manuscript. Toshihiro Misumi contributed to analysis and interpretation of data. Shigeru Aoki designed the study and assisted in the preparation of the manuscript. Hisashi Masuyama,Takeshi Nagamatsu, and Takeshi Umazume contributed to collect data. Astuo Itakura and Tomoaki Ikeda contributed to review the manuscript and final approval pf the version to be published.

## Supporting information


**Figure S1.** Friedman's curve for primiparous women.

**Figure S2.** Zhang's labor curve.

## Data Availability

Research data are not shared.
