# Supplementary figures and images for: Spontaneous labor curve based on a retrospective multi‐center study in Japan
Source: J Obstet Gynaecol Res. 2021 Oct 7;47(12):4263–9. doi: 10.1111/jog.15053 (PMC9291815; doi:10.1111/jog.15053)

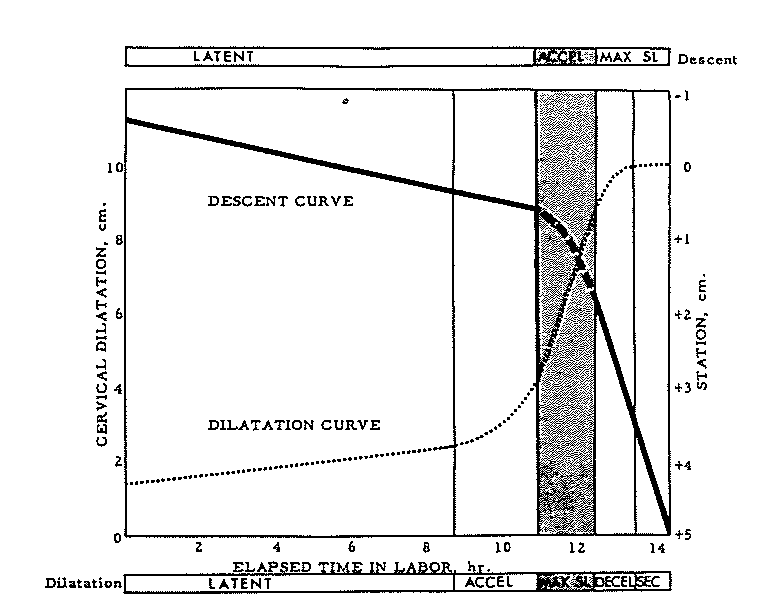

Supplement: Supplementary file 1 — Figure S1. Friedman's curve for primiparous women. [file JOG-47-4263-s001.tif]

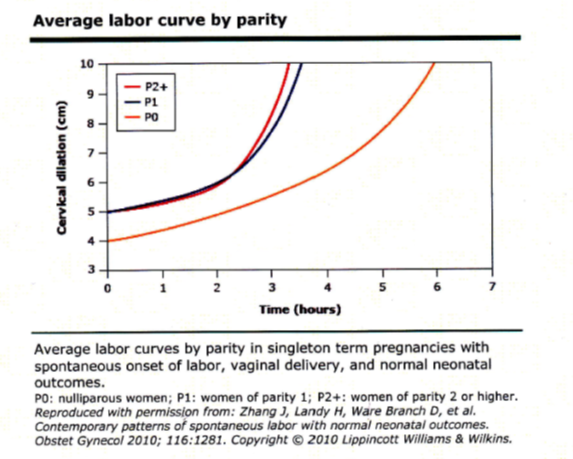

Supplement: Supplementary file 2 — Figure S2. Zhang's labor curve. [file JOG-47-4263-s002.png]
